# Hemophagocytic Lymphohistiocytosis in Association with Primary Cutaneous Anaplastic Large Cell Lymphoma

**DOI:** 10.1155/2014/384123

**Published:** 2014-10-28

**Authors:** Aneesh Basheer, Somanath Padhi, Ramesh Nagarajan, Vinoth Boopathy, Sudhagar Mookkappan, Nayyar Iqbal

**Affiliations:** Pondicherry Institute of Medical Sciences, Ganapathichettikulam, Pondicherry 605014, India

## Abstract

Hemophagocytic lymphohistiocytosis (HLH) has a well known association with lymphomas, especially of T cell origin. Prognosis of lymphoma associated HLH is very poor, especially in T cell lymphomas; and, therefore, early diagnosis might alter the outcome. Though association of HLH with systemic anaplastic large cell lymphoma (ALCL) is known, its occurrence in primary cutaneous ALCL (C-ALCL) is distinctly rare. We aim to describe a case of C-ALCL (anaplastic lymphoma kinase (ALK)−) in an elderly male who succumbed to the complication of associated HLH, which was possibly triggered by coexistent virus infection. We briefly present the literatures on lymphoma associated HLH and discuss the histopathological differentials of cutaneous CD30+ lymphoproliferative disorders. We do suggest that HLH may pose diagnostic challenges in the evaluation of an underlying lymphoma and hence warrants proper evaluation for the underlying etiologies and/or triggering factors.

## 1. Introduction

Hemophagocytic lymphohistiocytosis (HLH) is a rare, potentially underrecognized, hyperinflammatory syndrome that may be primary or secondary to several conditions such as infections (most common), inflammatory disorders, and hematological malignancies. Epstein Barr virus (EBV) is reported to be the most common risk factor implicated in the pathogenesis of HLH [1, 2]. Lymphoma associated HLH is the most common form of malignancy associated HLH [3]. Although HLH is known to complicate several subtypes of lymphomas, its association with T cell non-Hodgkin lymphoma (T-NHL) is reported to be most common; and occurrence among patients with B cell NHL (B-NHL), and Hodgkin lymphoma (HL) is rare [3–5].

Anaplastic large cell lymphoma (ALCL) is an uncommon form of mature T cell lymphoma which constitutes 2 to 3% of all NHLs. It is characterized by a polymorphous population of both neoplastic large pleomorphic cells (with an “embryo-like” or “horse-shoe” like nuclei (so-called Hallmark cell)) and reactive inflammatory cells, a tendency to invade lymphoid sinuses, and widespread expression of CD30 antigen (in greater than 75% of the tumor cells). Majority of these tumors are associated with a specific translocation *t* (2; 5) (p23; q35) resulting in the overexpression of the anaplastic lymphoma kinase (ALK) gene [6]. As per the 2008 WHO classification, ALCL encompasses 3 entities with overlapping histopathologic, but contrasting clinical and immunophenotypic characteristics, namely, systemic ALCL (ALK+), systemic ALCL (ALK−), and primary cutaneous ALCL (C-ALCL) [7]. There have been sporadic reports of HLH complicating the clinical course of ALCL, more so in systemic form of ALCL, rather than C-ALCL [8, 9].

In this report, we aim to describe a case of C-ALCL in an elderly male who succumbed to the complication of coexisting HLH, possibly triggered by underlying viral infection. Furthermore, we present a brief literature review on lymphoma associated HLH and discuss the diagnostic challenges in evaluation of coexistent HLH and lymphoma.

## 2. Case Presentation

A 56-year-old farmer presented to the out-patient department with complaints of high grade, intermittent fever, abdominal distension, and vomiting for 20 days. He also suffered from loss of appetite for past 5 days. He noticed a swelling over his left shoulder since past 5 days. There was no history of loss of weight, breathlessness, cough, or bone pain. Ten months prior to his present admission, he was evaluated in the same hospital for fever and jaundice and found to have a transudative pleural effusion and acalculous cholecystitis. Subsequently, he underwent laparoscopic cholecystectomy. He neither smoked nor consumed alcohol.

**Figure 1:**
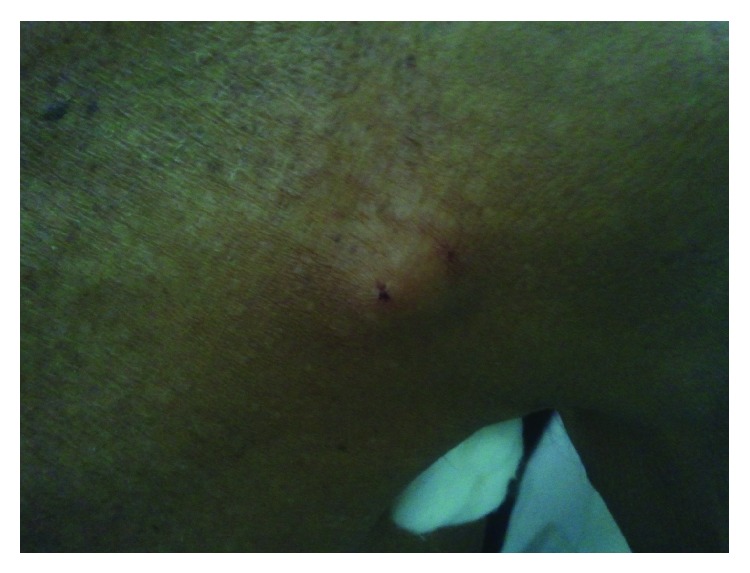
Swelling measuring 2 × 2 cm over the left shoulder. Swelling was hard and non-tender.

On examination, he was febrile. General physical examination revealed pallor, pan-digital clubbing, and bipedal edema. Besides, there were 2 nontender, hard, subcutaneous swellings, one measuring 2 × 2 cm on the left shoulder and the other 1 × 1 cm over the left anterior axillary line (Figure 1). Systemic evaluation revealed firm, nontender hepatomegaly (liver span, 16 cm) and firm splenomegaly (4 cm below the left costal margin), and no significant peripheral lymphadenopathy. There were no stigmata of chronic liver disease. Disseminated tuberculosis, human immunodeficiency virus (HIV) infection, disseminated malignancy (lymphomas/leukemia), and connective tissue disorders were among the differential diagnoses considered.

Laboratory investigations showed normocytic normochromic anemia (hemoglobin (Hb), 84 g/L (reference, 120–140 g/L)), leukopenia (total leucocyte count (TLC), 2 × 10^9^/L (reference, 4–11 × 10^9^/L)), differential of neutrophil, 49%, lymphocyte, 49%, and monocytes, 2%, absolute neutrophil count (ANC), 980/cmm, total platelet count (TPC), 175 × 10^9^/L (reference, 150–450 × 10^9^/L), and no atypical cells/blasts. His biochemical parameters revealed elevated serum aspartate aminotransferase/SGOT (546 U/L, reference, 15–41 U/L), alanine aminotransferase/SGPT (208 U/L, reference, 10–40 U/L), alkaline phosphatase (701 U/L, reference, 38–126 U/L), lactate dehydrogenase (1094 U/L, reference, <300 U/L), ferritin (2400 ng/mL, reference, 25–340 ng/mL), and vitamin B_12_ (>2000 pg/mL, reference, 191–890 pg/mL). His complete microbiological work-up for possible infectious organisms and serologic tests for HIV, HBV, HCV, ANA, and antidouble stranded DNA were negative. Complete radiological evaluation revealed hepatosplenomegaly (without any focal lesions), mild ascites, and bilateral pleural effusion.

**Figure 2:**
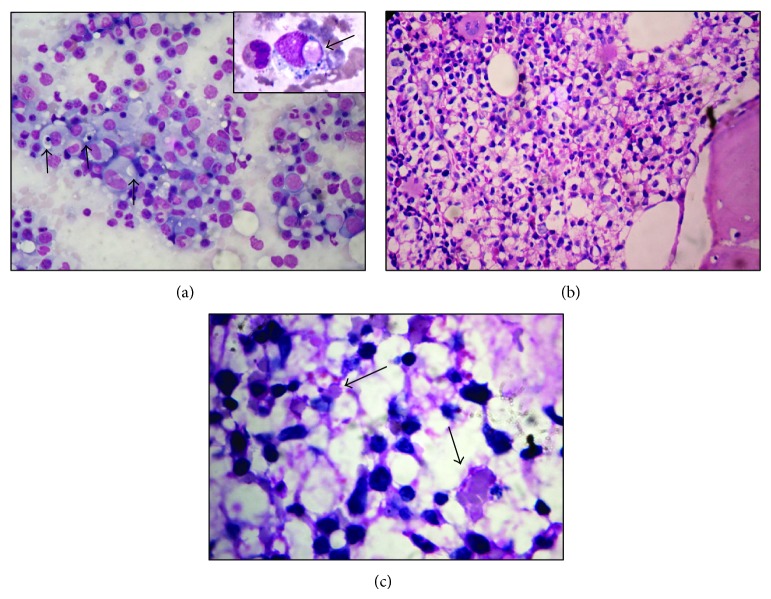
Bone marrow aspirate smear (a) showing hypercellularity and increased population of benign histiocytes with evidence of haemophagocytosis (black arrows, 200x). Note the evidence of erythrophagocytosis by the histiocytes (inbox, black arrow, 400x) (May Grunewald Giemsa stain). Bone marrow trephine biopsy section showing increased population of benign histiocytes in the intertrabecular marrow space (b, haematoxylin eosin, 200x). These histiocytes showed engulfed debris and obvious erythrophagocytosis (c, black arrow) (haematoxylin eosin, 400x).

**Figure 3:**
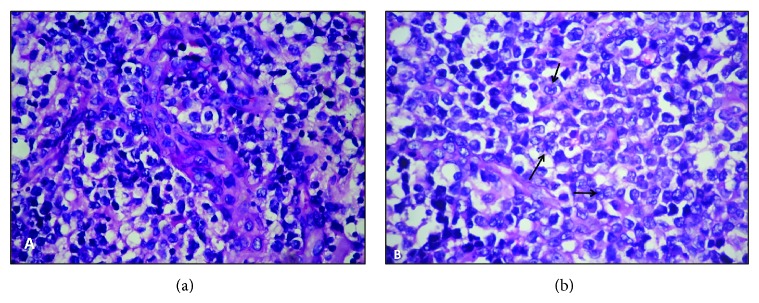
Paraffin embedded tissue sections from the excised left shoulder nodule showing sheets and aggregates of pleomorphic tumor cells with irregular nuclear contour and prominent nucleoli. The tumor cells showed angiocentricity (a) and at places, “embryo”/“horse shoe” like nuclei, prominent nucleoli (so-called “hallmark” cells) ((b), black arrows) (haematoxylin eosin, 400x).

**Figure 4:**
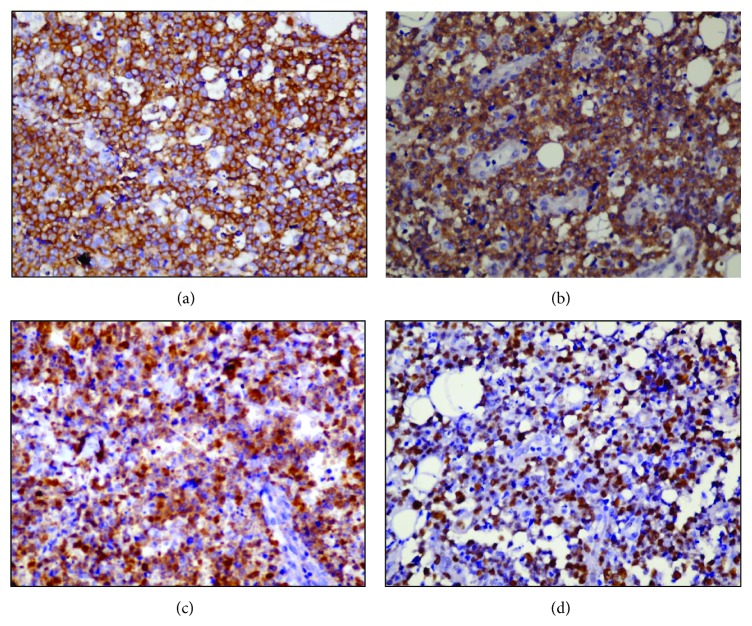
On immunohistochemistry the tumor cells showed uniform intense positivity for leukocyte common antigen (LCA) (a), CD3 (b), and CD30 (c), with a high proliferation index (Ki-67 = 70%) (d). Note the uniform, strong Golgi region positivity of CD30 (c) in the majority (>75%) of tumor cells. The cells were negative for pancytokeratin, CD20, anaplastic large cell lymphoma kinase (ALK), and epithelial membrane antigen (EMA) (Peroxidase-antiperoxidase stain, a and b; 100x, c; 200x).

Patient continued to have spiking fever, and, in view of persistent bicytopenia, bone marrow aspiration and biopsy were done, which showed increased population of benign histiocytes with evidence of hemophagocytosis. There was no evidence of any granuloma, hemoparasites, or malignancy. Histopathological evaluation of the excised left shoulder nodule revealed a high grade tumor in the dermis with large, nonepidermotropic, pleomorphic cells with “horse-shoe” shaped nuclei and prominent nucleoli. There was evidence of vascular proliferation and the tumor cells exhibited angiocentricity. Further characterization using immunohistochemistry (IHC) showed that the tumor cells were positive for leucocyte common antigen (LCA), CD3, and CD30 (uniform diffuse positivity in Golgi region in more than 75% of cells) but negative for pan cytokeratin, CD20, ALK, and epithelial membrane antigen (EMA). The tumor cells showed a higher proliferation index (Ki-67 = 70%). The histopathological features, in correlation with IHC characteristics, were consistent with the diagnosis of ALCL (CD30+, ALK−) (Figures 2(a), 2(b), 2(c), 3(a), 3(b), 4(a), 4(b), 4(c), and 4(d)). In view of the domainant cutaneous presentation, lack of significant lymphadenopathy and absence of obvious bone marrow infiltration (CD 30 negative), the diagnosis of C-ALCL with associated HLH at presentation was rendered. The patient was managed with steroids and ongoing broad spectrum intravenous antibiotics, and hematologist's opinion was taken. However, patient succumbed to disseminated intravascular coagulation (DIC) and multiorgan dysfunction, secondary to HLH, on 5th day of diagnosis of the HLH and lymphoma (11th day post admission). The informed consent was obtained from the next of kin of the patient.

## 3. Discussion

The hallmark of HLH is phagocytosis of mature blood elements (red cells, leucocytes, and platelets) or their precursors by benign macrophages throughout the reticuloendothelial system secondary to immune dysregulation and excessive triggering of cytokine production. In the absence of a positive family history or a documented genetic/molecular abnormality, the diagnosis of HLH requires the presence of at least, five of eight HLH-2004 criteria put forth by the HLH society [10]. The diagnosis of lymphoma associated HLH requires the same criteria in addition to the documented histological evidence of a lymphoma [3]. Our case satisfied the required criteria (5/6 tested) for the diagnosis of HLH such as persistent high grade fever, bicytopenia (Hb < 90 g/L, ANC < 1000/cmm), splenomegaly in the absence of focal lesions, hyperferritinemia, and histiocytic hemophagocytosis in the bone marrow. In addition to HLH, there was a documented evidence of a lymphoma in our patient.

A comprehensive review on lymphoma associated HLH is presented in Table 1. Compared to B cell NHLs and HLs, T cell NHLs are frequently reported to be complicated with HLH during their course of the disease; and this confers a worse prognosis [4, 5, 11–14]. Lymphomas associated with HLH are more often reported among males than females. The median age at presentation/diagnosis for T-NHL and HL associated HLH has been reported to be 45 years, whereas B-NHL associated HLH has been frequently reported among elderly population (median age = 6th decade) [3, 12, 14]. Peripheral T cell lymphoma, not otherwise specified (PTCL-NOS), extranodal NK/T cell lymphomas (EN-NKTCL), subcutaneous panniculitis-like T cell lymphoma (SCPTCL), angioimmunoblastic T cell lymphoma (AITL), and rarely ALCL are more likely to be complicated by secondary HLH [4, 7, 11–14]. Among B-NHLs, diffuse large B cell lymphoma (DLBCL) is the most common histological subtype implicated in HLH. Among HL, lymphocyte depleted and mixed cellularity subtypes have been reported to be associated with HLH [5]. Several large population based studies among Asian patients have found that T-NHL associated HLH is characterized by (i) advanced stage at presentation, (ii) frequent occurrence of B symptoms, organomegaly (and lymphadenopathy), cytopenia (s), liver dysfunction, and coagulopathy, (iii) higher proportion of bone marrow infiltration, and (iv) markedly reduced overall survival with or without definitive chemotherapy. Several biochemical parameters such as raised *β*_2_ microglobulin (>2200 mcg/L), CA-125 (>35 IU/L), ferritin, fasting triglyceride, and lactate dehydrogenase and reduced fibrinogen have been reported to be significantly (*P* < 0.001) associated with HLH subgroups of T-NHL and have been suggested to be important biomarkers of T-NHL associated HLH [4, 11–13]. On the contrary, B-NHL associated HLH has been reported to be associated with lesser degree of bone marrow involvement, significant lack of associated lymphadenopathy, a higher propensity for intravascular lymphomatosis, higher association with EBV, and overall better survival following chemotherapy [3, 14]. The largest French study (to date) on HL associated HLH (*N* = 34, 8/34; HIV+ve) reported that 100% of these patients were at advanced stage (IVB), and there was a strong association (32/34, 92%) with EBV detected by either immunohistochemistry or in situ hybridization technique [5]. The authors of that study suggested that high levels of latent membrane protein (LMP)-1 expression may induce the large amount of Th_1_ cytokines production by Reed Sternberg cells in HL leading to HLH.

Primary cutaneous ALCL is the second most common form of primary cutaneous T cell lymphoma after mycoses fungoides and carries an excellent prognosis with 5-year overall survival of nearly 90% following surgical excision and/or local radiation therapy. Though it presents as a solitary ulcerated nodule, multifocal involvement may be seen in less than 20% of cases. Along with benign/reactive lymphomatoid papulosis (LyP), it constitutes the spectrum of primary CD30 positive lymphoproliferative disorders of the skin. However, this needs to be differentiated from the more common cutaneous involvement in both PTCL-NOS and systemic ALCL (ALK−) by means of appropriate ancillary studies [6, 7]. Simultaneous occurrence of HLH and lymphoma in our patient led to a diagnostic dilemma with more common PTCL-NOS and/or systemic ALCL (ALK−) with dominant cutaneous involvement. However, our patient had following characteristics which favored a final diagnosis of C-ALCL over systemic ALCL (ALK−)/PTCL-NOS. These include (i) older age at presentation, (ii) dominant cutaneous involvement in the absence of demonstrable involvement of bone marrow, (iii) lack of significant peripheral lymphadenopathy, and (iv) ALK negative, intense, and Golgi zone CD30 positivity in >75% tumor cells (in contrast to focal positivity in PTCL-NOS) [7]. Furthermore, only few specific markers such as nuclear survivin expression (absent in C-ALCL, positive in systemic ALCL) and punctuate clusterin expression (seen in C-ALCL) may help in differentiating C-ALCL from systemic ALCL with dominant cutaneous involvement [6]. These markers were not used in our case due to non availability in our center. Considering the favorable outcome in C-ALCL compared to its systemic counterpart, we presume that the fatal outcome in our patient was more likely the result of complication of HLH, rather than the C-ALCL itself. This probably was the result of an underlying triggering event such as infection with EBV, Parvo-B19, or cytomegalo virus which unfortunately was not tested in our patient due to financial and time constraint.

To conclude, dominant presentation of HLH may overshadow the underlying lymphoma creating great diagnostic and therapeutic challenges for the treating physician. Therefore, a high index of suspicion, early diagnosis, and early institution of therapy might alter the outcome in these patients.

## Figures and Tables

**Table 1:** Comprehensive review on lymphoma associated hemophagocytic lymphohistiocytosis (HLH).

		With HLH (*N* = 36)	Without HLH (*N* = 123)	*P* value	Remark

Xie et al. [4], *N* = 159 (peripheral T cell lymphoma)	Age (years), range, mean	16–78 (48)	10–77 (46)	—	Most common histological types, PTCL-NOS Less Common types, AITL, SCPTCL, NKTCL; prognosis is poor in HLH group. *High β*_2_ * microglobulin, CA-125 are important biomarkers in T-NHL associated HLH*.
	Male/female	23/13	86/17	—	
	Stage III/IV, number, %	34/36 (94%)	93/123 (76%)	0.013	
	B symptoms	100%	76%	0.001	
	Fever	92%	43%	<0.001	
	Organomegaly	72%	23%	<0.001	
	Bicytopenia	78%	13%	<0.001	
	Bone marrow involvement	56%	26%	<0.002	
	Median survival	3 months	16 months	—	
	Overall survival (log rank test)	11%	44%	<0.001	
	Biochemical parameters				
	*β*_2_ microglobulin (>2200 mcg/L)				
	Ferritin (>500 pg/mL)				
	Fibrinogen (<1.5 g/L)				
	Liver dysfunction	More common	Less common	<0.003	
	CA-125 (>35 IU/L)				
	Triglyceride (>265 mg/dL)			0.015	
	Lactate dehydrogenase (>225 mg/dL)			>0.05	

		With HLH	Without HLH	*P* < 0.05	Remark

Tong et al. [11] *N* = 113 (T cell NHL)	Number (*N*)	28/113	85/113		Fever, raised LDH, ferritin, CA-125, *β*_2_ microglobulin, hepatosplenomegaly, cytopenias are more in favor of T cell lymphoma associated HLH.
	Elevated lactate dehydrogenase	100%	55%		
	High ferritin	100%	64%		
	Fasting hypertriglyceridemia	79%	43%		
	Hypofibrinogenemia	43%	14%		
	Bone marrow involvement	57%	32%		
	Liver dysfunction	40%	13%		
	Median survival	40 days	8 months		

Han et al. [12] (*N* = 29, lymphoma associated HLH)	*N* = 29 Male/female, 16/13 Age, 46 years (9–87) 14, high-intermediate IPI, 12, high IPI	Neutropenia, 11/29 (37.9%), thrombocytopenia, 25/29 (86.2%), Hb < 90 g/L, 16/29 (55.2%), ferritin >2000 pg/mL, 13/14, LDH > 500 IU/L, 26/26, low fibrinogen, 15/26 (57.7%), DIC, 16/29. Histological subtypes: EN-NKTCL (37.9%), PTCL-NOS (27.6%), DLBCL (17.2%), ALCL (ALK-) (6.9%).			Median survival = 36 days DIC, jaundice, T/NK cell phenotype; poor clinical response-bad markers. ***12/17; EBV positive***.

	With bone marrow involvement (*N* = 70)	Without bone marrow involvement (*N* = 103)		*P* value	Remark

Tong et al. [13] (*N* = 173, peripheral T cell NHL)	Lymphoma associated HLH, 25/70 (36%)	Lymphoma associated HLH, 8/103 (8%)		<0.001	Common histological subtypes with bone marrow involvement: AITCL, PTCL-NOS, ALCL, EN-NKTCL, EATCL
	High ferritin and liver dysfunction: common	Less common		<0.05	
	Anemia: 51%	29%		0.001	
	1 year overall survival: 5%	49%		

Ménard et al. [5] (*N* = 34, Hodgkin lymphoma associated HLH)	Males/females, 26/6, M : F = 3.3 : 1 Age = 43 years (19–84 years), 26, HIV nonreactive, 8, HIV +ve. 100%, stage IVB	9/20 (45%), lymphocyte depleted, 8/20 (40%), mixed cellularity, 3/20 (15%), nodular sclerosis subtype 32/34 (92%) cases +ve for EBER (ISH), LMP-1 (IHC), ZEBRA −ve in all			***HL-HLH strongly associated with EBV***. Prognosis not studied. High levels of LMP-1 expression may induce the large amount of Th1 cytokines production by Reed Sternberg cells leading to HLH.

			B-NHL-HLH	NKTCL-HLH	Remark

Takahashi et al. [14], Japan, (*N* = 142, lymphoma associated HLH)	Number (*N*) Median age at diagnosis Cytopenia(s), coagulopathy, liver dysfunction Intravascular lymphomatosis Hepatosplenomegaly Lymphadenopathy **EBV positivity (ISH)** Median survival (days)		68 63.5 years less common 10/20 + Nil **3/24** 242	74 49 year more common — + + **19/23** 69	Prognosis is better in B-NHL associated HLH than NKTCL associated HLH.

HLH: hemophagocytic lymphohistiocytosis, PTCL-NOS: peripheral T cell lymphoma, not otherwise specified, AITL: angioimmunoblastic T cell lymphoma, SCPTCL: subcutaneous panniculitis-like T cell lymphoma, EN-NKTCL: extranodal natural killer/T cell lymphoma, T-NHL: T cell non-Hodgkin lymphoma, EATCL: enteropathy associated T cell lymphoma, ALCL: anaplastic large cell lymphoma, ALK: anaplastic lymphoma kinase, DLBCL: diffuse large B cell non-Hodgkin lymphoma, not otherwise specified, B-NHL-HLH: B cell non-Hodgkin lymphoma associated HLH, NKTCL-HLH: natural killer cell T cell lymphoma associated HLH, IPI: international prognostic index, LDH: lactate dehydrogenase, DIC: disseminated intravascular coagulation, EBV: Epstein Barr virus, EBER: Epstein Barr encoding region, ISH: in situ hybridization, LMP: latent membrane protein, and IHC: immunohistochemistry.
